# How Invariant Feature Selectivity Is Achieved in Cortex

**DOI:** 10.3389/fnsyn.2016.00026

**Published:** 2016-08-23

**Authors:** Tatyana O. Sharpee

**Affiliations:** Computational Neurobiology Laboratory, Salk Institute for Biological StudiesLa Jolla, CA, USA

**Keywords:** object recognition, Convolutional Neural Networks (CNN), visual system, area V4, phase invariance, quadrature model, curvature, auditory system

## Abstract

Parsing the visual scene into objects is paramount to survival. Yet, how this is accomplished by the nervous system remains largely unknown, even in the comparatively well understood visual system. It is especially unclear how detailed peripheral signal representations are transformed into the object-oriented representations that are independent of object position and are provided by the final stages of visual processing. This perspective discusses advances in computational algorithms for fitting large-scale models that make it possible to reconstruct the intermediate steps of visual processing based on neural responses to natural stimuli. In particular, it is now possible to characterize how different types of position invariance, such as local (also known as phase invariance) and more global, are interleaved with nonlinear operations to allow for coding of curved contours. Neurons in the mid-level visual area V4 exhibit selectivity to pairs of even- and odd-symmetric profiles along curved contours. Such pairing is reminiscent of the response properties of complex cells in the primary visual cortex (V1) and suggests specific ways in which V1 signals are transformed within subsequent visual cortical areas. These examples illustrate that large-scale models fitted to neural responses to natural stimuli can provide generative models of successive stages of sensory processing.

The current predominant hypothesis is that robust object recognition is made possible by transforming detailed signal representations to representations that encode objects independent of the viewing position (DiCarlo and Cox, 2007; Serre et al., 2007; DiCarlo et al., 2012). Such object-centered representations make it possible to perform fine discrimination, because these representations combine signals from viewing conditions wherein two objects might appear similar and where they are easily distinguishable. Achieving such object-centered representations is not trivial because in most cases integration across viewing positions destroys the specificity to configuration of object parts that is essential for correct identification (Ullman and Soloviev, 1999). Empirical studies in computer vision emphasize that increases in, for example, position tolerance have to be gradual and have to be interleaved with increases in specificity to the more complex features that will ultimately make it possible to distinguish between different objects (Ullman and Soloviev, 1999). How these representations are built in the visual system remains largely unknown. Similar computational tasks need to be solved by other sensory systems, including the somatosensory (Maravall and Diamond, 2014) and auditory systems (King and Nelken, 2009; Theunissen and Elie, 2014). Specifically, auditory perception includes a tolerance to changes in loudness, cadence and pitch (Trefethen and Embree, 2005). Again, however, the details of signal transformations within the auditory system remain to be worked out.

In this regard, large-scale models can provide vital information about how signals are transformed across their sensory processing pathways. So far, we know that neurons in early stages of cortical processing are primarily driven by simple stimulus features. Examples of such features include edges, in the case of neurons in the primary visual cortex (V1), and analogous features in the space of spectrotemporal modulations for neurons in primary auditory cortex or its analog in birds (Nagel and Doupe, 2008; Theunissen and Elie, 2014). Neurons at later stages tend to be selective for more complex combinations of stimulus features (Connor et al., 2007). For example, neurons in the mid-level visual area V4 exhibit selectivity for contour curvature (Connor et al., 2007). Neurons at subsequent stages of visual processing, such as in the inferotemporal (IT) cortex, exhibit selectivity for faces and their components (Tsao and Livingstone, 2008), as well as other objects of large biological significance (Desimone et al., 1984). Concomitant with the complexity of image features that drive the responses of visual neurons from V1 to V4 to IT, there is also an increase in the degree of tolerance that the responses of these neurons exhibit when relevant image features are displaced or scaled in size (DiCarlo et al., 2012; Roe et al., 2012). Importantly, artificial neural networks with this general structure can be optimized to reach human levels of categorization performance on a variety of visual recognition tasks (Khaligh-Razavi and Kriegeskorte, 2014; Yamins et al., 2014). Thus, different sensory systems are all organized hierarchically with a progressive increase in the invariance and selectivity of neural responses to complex stimulus features (Connor et al., 2007; Meliza and Margoliash, 2010; DiCarlo et al., 2012; Roe et al., 2012). Nevertheless, the specific routes that signals take within the mid-and high-level sensory areas are difficult to characterize because they involve multiple intermediate nonlinear transformations and an incredible degree of convergence across brain regions. For example, some estimates suggest that a single neuron in area V4 can combine signals that originate from a substantial fraction of the V1 surface (Motter, 2009). If this pooling were indiscriminative, without any guiding principles, then this would seem to preclude any functional object recognition thought to be mediated by these brain regions (DiCarlo et al., 2012; Roe et al., 2012). Complicating the matter further, the process of feature extraction is a dynamic process (Olshausen et al., 1995) that is affected by neural adaptation to stimulus statistics (Sharpee et al., 2006; McManus et al., 2011) as well as by cognitive tasks, such as attention and perceptual learning (Ito et al., 1998; Ito and Gilbert, 1999).

Despite the difficulties, some progress can be made by fitting neural responses with multi-scale computational models that use built-in constraints to reduce the number of parameters incurred when characterizing the feature selectivity of mid- and high-level sensory neurons. For visual neurons, position invariance is one of the dominant constraints (Bouvrie et al., 2009; Lee et al., 2009). Models that incorporate position invariance explicitly are known as hierarchical convolution networks (Le Cun et al., 1989; Khaligh-Razavi and Kriegeskorte, 2014; Yamins et al., 2014; Vintch et al., 2015). Such models achieve good performance on the object recognition task. However, the computations performed by the optimized models are difficult to interpret (Yamins and DiCarlo, 2016). To circumvent this problem, one can develop methods that explicitly determine the features that drive the responses of any given neuron while simultaneously taking into account position invariance (Eickenberg et al., 2012; Sharpee et al., 2013; Zeiler and Fergus, 2014; Vintch et al., 2015). The corresponding model is schematically depicted in Figure 1A. Compared to standard models that estimate relevant features without position invariance (de Boer and Kuyper, 1968; Victor and Shapley, 1980; Chichilnisky, 2001; Nykamp and Ringach, 2002; Bialek and de Ruyter van Steveninck, 2005; Schwartz et al., 2006; Fitzgerald et al., 2011), convolutional models drastically reduce the number of independent parameters when they require the relevant image features to be the same for each position within the neuron's response field. Although the model estimates only two relevant image features per position, when pooling across positions is taken into account, this is equivalent to estimating models with as many as ~50–100 relevant image features. With such a reduction in model complexity, it becomes feasible to begin deciphering how neural circuits simultaneously achieve invariance and selectivity for complex stimulus features.

**Figure 1:**
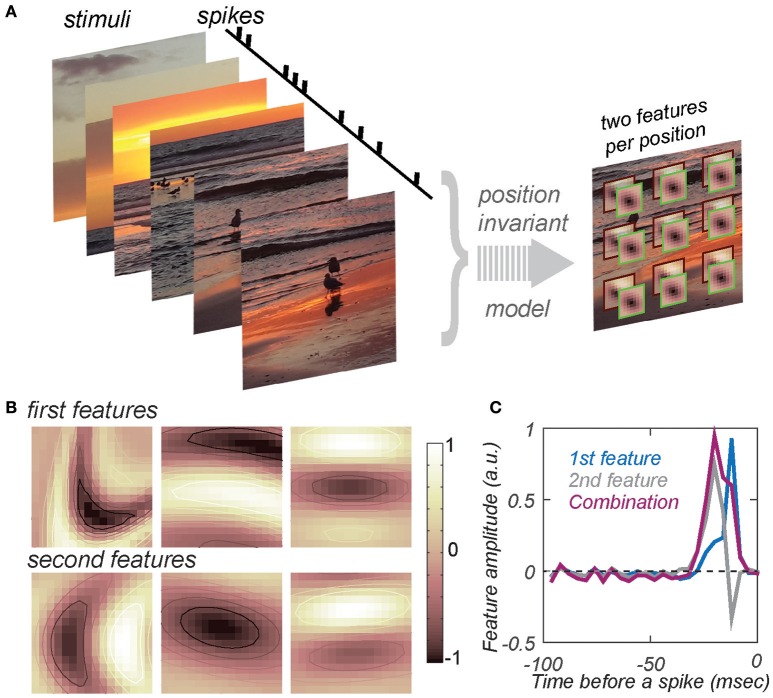
**Estimating feature selectivity and invariance properties from neural responses to natural stimuli. (A)** Schematic of the model that combines position invariance with selectivity to conjunctions of two features at one position in the visual space. At each position in the visual space, stimuli are compared to the two features. The result of this computation produces two projection values, one for each feature. The two projection values are combined according to a nonlinear function (determined by fitting and not shown here). The results of this nonlinear computation at each position are combined according to a MAX (or logical OR) operation to obtain a prediction for the spike rate elicited by the stimulus. **(B)** Examples of the two most relevant image features for three V4 neurons from two different animals. Columns refer to different neurons, from left to right: m26a_3, j15c_1, j46a_1. The first and second rows show the first and second maximally informative feature per neuron, respectively. Each feature is shown after fitting by a curved Gabor model to the templates estimated from the responses of these neurons to natural stimuli (Sharpee et al., 2013). **(C)** A pair of two most relevant temporal profiles for an auditory neuron. Data are from field L (Sharpee et al., 2011a), a region analogous to the mammalian primary auditory cortex (Sharpee et al., 2011a). The sum of the two relevant features (magenta) produces a time dilated version of the first feature (blue). Neuron “udon2120.”

Models of this structure have recently been used to describe how neurons in the mid-level visual area V4 encode natural stimuli (Sharpee et al., 2013). One of the concerns when fitting such models is that the stimulus set needs to be diverse enough to probe different aspects of the neural response. Stimuli from the natural sensory environment fulfill this requirement. Natural stimuli also elicit robust responses of neurons at different stages of sensory processing. In particular, mid- and high-level sensory neurons exhibit stronger responses when exposed to natural stimuli as compared to randomized inputs (Sen et al., 2001). Historically, randomized stimuli have primarily been used to characterize neural feature selectivity because they allow for computationally simpler estimation procedures (Bialek and de Ruyter van Steveninck, 2005; Gollisch, 2006; Schwartz et al., 2006; Dimitrov et al., 2009; Samengo and Gollisch, 2012). However, the increased availability of computing resources now makes estimation procedures tenable with natural stimuli. A typical dataset of responses from an individual neuron includes ~300 movie segments as stimuli, each containing ~100 frames updated at 30 Hz. (The duration of individual movie segments is limited by how long animals can maintain fixation in awake experiments). Thus, models are typically fit using ~30,000 stimulus/response associations. Such large numbers of stimuli are needed in order to probe the neural response function under a broad range of conditions, and because it is not known *a priori* which movie segment will elicit a high firing rate from a given neuron. The fitting of the model used in Figure 1A to the responses of individual neurons produces (1) a pair of most relevant image features for a neuron, (2) the nonlinear function describing how these two features jointly affect its neural response, and (3) the range of position invariance, defined as the range of positions in the visual space across which signals are combined according to logical OR or Max operations (Figure 1A). All of these parameters can be estimated through maximum likelihood fitting (or related methods) based on neural responses to a large set of stimuli. The position invariance can be modeled either with uniform (Sharpee et al., 2013) or graded (Vintch et al., 2012) contributions across positions to the measured neural response. Once a convolutional model is fitted to the responses of a set of recorded neurons, the distribution of its parameters also produces a so-called generative model (Yamins and DiCarlo, 2016). Generative models are those that can recreate a set of responses across a neural population, yielding a distribution of parameters that best characterize the feature selectivity and invariance ranges. This distribution is obtained by fitting the model to a set of recorded neurons. With this setup, we can now discuss what convolutional models have revealed about the distribution of these parameters in mid-level visual area V4.

## Curvature selectivity

The reconstructions of V4 neural responses to natural stimuli indicate selectivity to segments of curve contours (Sharpee et al., 2013); examples of the relevant features are given in Figure 1B. Obtained with natural stimuli, this observation extends previous reports of curvature selectivity obtained with curved parametric stimuli (Gallant et al., 1996; Pasupathy and Connor, 1999, 2001, 2002; David et al., 2006; Connor et al., 2007) to the case of more diverse stimulus conditions. It is worth noting that natural stimuli were optimized neither for curvature selectivity nor for a particular neuron or area. In fact, the same set of stimuli when used in V1 yields selectivity to straight contours (Sharpee et al., 2006, 2008). This insures that any curvature selectivity obtained by analyzing V4 responses reflects genuine aspects of their feature selectivity that are not influenced by the stimulus properties themselves. Furthermore, the fact that curvature selectivity also appears for mid-level units in artificial networks after optimization to maximize natural stimuli classification (Cadieu and Olshausen, 2012; Zeiler and Fergus, 2014) reinforces the notion that neural circuits are optimized for the structure of the natural sensory environment (Bialek, 2013).

## Invariance-complexity trade-off

The tightness of the preferred contour's curvature (in what follows we will refer to it as the preferred curvature value) varies substantially across neurons (Sharpee et al., 2013; Figure 1B). Intuitively, tighter curvatures can be viewed as describing more complex relevant image features compared to more shallow or straight contours. Supporting this intuition, tighter curvatures are also less frequently observed in natural scenes as compared to contours with more shallow curvatures (Lawlor and Zucker, 2013; Sharpee et al., 2013). Given that invariance and complexity concomitantly increase from stage to stage within the ventral visual pathway that performs visual object recognition, one might have expected that contours with tighter curvatures would be associated with larger ranges of position invariance. In this regard, the second observation came as a surprise because the opposite trend was observed by (Sharpee et al., 2013): neurons with smaller ranges of position invariance had preferred image features with tighter curvatures. This trend was reproduced in experiments with parametric stimuli (Nandy et al., 2013). It is also congruent with recent reports on the trade-off between position invariance and selectivity (Cadieu et al., 2007; Zoccolan et al., 2007; Rust and Dicarlo, 2010; Rust and DiCarlo, 2012). The preference of neurons with smaller ranges of position invariance for more tightly curved contours, together with the reduced frequency of curved contours in the natural environment, could explain at least in part the observed trade-off between invariance and selectivity (Rust and Dicarlo, 2010; Rust and DiCarlo, 2012).

## Phase or local position invariance in V4

Some convolutional models make it possible to estimate conjunctions of features that simultaneously affect the neural response at each retinotopic position (Eickenberg et al., 2012; Sharpee et al., 2013; Vintch et al., 2015). Applying these methods to V4 responses to natural stimuli, one finds that the two most relevant features of a given neuron often formed a pair of odd- and even-symmetric functions in the direction perpendicular to the preferred contour. This type of selectivity is reminiscent of the selectivity established for V1 complex cells (Adelson and Bergen, 1985). In V1, the so-called energy model accounts for the responses of V1 neurons as a quadratic function of the output of two relevant features: an even (cosine) and odd (sine) function in the direction perpendicular to the preferred orientation of the V1 neuron. With probed with grating stimuli, the output of this model does not vary with the phase of the grating. For this reason, this type of selectivity to combinations of even- and odd-symmetric functions also became known as phase invariance. However, phase invariance also corresponds to local position invariance. This is because the odd-symmetric function can be well approximated as the difference of two slightly displaced even-symmetric functions (think of an edge as the difference between two bars). Thus, a model that allows for multiple relevant features can account for local position invariance even if does not have explicit convolutional architecture.

These arguments can now help interpret the results obtained in V4 using convolutional models (Sharpee et al., 2013). The convolutional model used in that study included only one explicit pooling stage. However, at each position, the two estimated most relevant features turned out to form a quadrature pair. This type of local feature selectivity indicates the presence of a local position invariance that is in addition to the more global position invariance captured by the convolutional part of the model. One important aspect of the quadrature pair selectivity observed locally in V4 is that it occurs with respect to curved contours, whereas in V1 it is observed with respect to straight contours. The most straightforward way of connecting these observations to the circuitry of the ventral visual pathway is to suppose that local position invariance corresponds to a summation of subunits representing V1 complex cells. This summation first takes place across different orientations, giving rise to curve contours, and then across positions, giving rise to positional invariance. Knowing that signals reach area V4 primarily through area V2, one could associate the second summation with a pooling of signals across V2 subunits. Further, the observed trade-off between preferred curvature and (global) invariance range (Sharpee et al., 2013) suggests that, for individual neurons, either a summation across orientations or across positions dominates.

The concept of local and global invariance is also directly applicable to other sensory circuits. For example, for the case of motion perception, neurons that project from V1 to MT are predominantly complex and orientation tuned (Movshon and Newsome, 1996). Given that MT neurons have 10 times larger receptive fields than the V1 complex cells whose responses they integrate (Simoncelli and Heeger, 1998), the responses of MT neurons would also be well described by a combination of local and global invariance.

In the auditory system, recent psychophysical studies found that birds attend to a mixture of local and global rhythmic features (Ten Cate et al., 2016). A re-examination of published neurophysiological data (Sharpee et al., 2011a) from field L, an area in bird's brain analogous to the mammalian primary auditory cortex, provides evidence for local invariance with respect to changes in cadence or time dilation in the responses of these neurons. Previous analyses showed that for neurons tuned to a specific frequency, the temporal profiles of the two most relevant features form a quadrature pair in a sense that they are described by a pair of integration/differentiation features (Sharpee et al., 2011a). This type of selectivity could be consistent with shifts in temporal offsets or temporal jitter (Aldworth et al., 2005; Dimitrov and Gedeon, 2006; Gollisch, 2006; Dimitrov et al., 2009) as well as with changes in cadence or time dilation. However, detailed statistical analysis ruled out temporal jitter as the cause underlying integration/differentiation pair of features for that dataset (Sharpee et al., 2011a). Furthermore, for auditory signals, integration over temporal latencies would only be relevant in the context of binaural time differences, which were not analyzed in Sharpee et al. (2011a). On the other hand, an integration over different time dilation would be perceptually relevant (Nagel et al., 2010) and would result in pairs of features that could also be approximated as integration/differentiation. Indeed, in the case of selectivity to the temporal profile *F(t)* and its time-dilated version *F(t/*τ*)*, for τ ~*1*, one would expect to find a combination of features *F(t)* and *F*′*(t)t*. If *F(t)* has unimodal shape, the second feature would approximate a time derivative. Figure 1C shows how a pair of integration/differentiation relevant temporal profiles for an auditory neuron can produce two unimodal features, one of which is a time-dilated version of the other. It is worth noting that similar types of selectivity were observed in the peripheral olfactory system (Kim et al., 2011) as well as in the granular layers of A1 cortex (Atencio et al., 2008, 2009). Neurons in the infragranular of A1 cortex exhibited more complex forms of selectivity, potentially analogous to the curvature selectivity discussed here for V4 neurons (Atencio et al., 2009; Sharpee et al., 2011b). The relationship between the two most relevant features of A1 neurons with such complex forms of auditory selectivity could potentially be consistent with a model of local invariance with respect to dilation in time or frequency, but this hypothesis would need to be quantitatively tested in future work.

Overall, recent progress in experimental and computational methods for fitting large-scale models to neural responses to natural stimuli offers the hope of reconstructing detailed transformations that make biological vision so much more efficient than machine vision. Clearly, the present models lack many of the important aspects of visual processing, including various forms of gain control (Carandini and Heeger, 2013), adaptive properties (Olshausen et al., 1995; Wark et al., 2007; McManus et al., 2011), and modulation by attention and cognitive tasks (Koch and Ullman, 1985; Olshausen et al., 1993; Ito et al., 1998; Ito and Gilbert, 1999). Increasingly more sophisticated models has been built for the retina that can relate better to the underlying neural circuitry (Kaardal et al., 2013; Freeman et al., 2015). Further, improvements in computational methods are needed to be able to scale and fit such detailed models to cortical responses.

## Author contributions

The author confirms being the sole contributor of this work and approved it for publication.

## Funding

This research was supported by the National Science Foundation (NSF) CAREER award number 1254123 and IOS-1556388, the National Eye Institute of the National Institutes of Health under Award Number R01EY019493, NEI Core grant P30EY019005, and T32EY020503, McKnight Scholarship, Ray Thomas Edwards Career Award, and the Janelia Visitor Scientist Program.

### Conflict of interest statement

The author declares that the research was conducted in the absence of any commercial or financial relationships that could be construed as a potential conflict of interest.
